# Artesunate promotes the proliferation of neural stem/progenitor cells and alleviates Ischemia-reperfusion Injury through PI3K/Akt/FOXO-3a/p27^kip1^ signaling pathway

**DOI:** 10.18632/aging.103121

**Published:** 2020-05-07

**Authors:** Kaiyuan Zhang, Yang Yang, Hongfei Ge, Ju Wang, Xuezhu Chen, Xuejiao Lei, Jun Zhong, Chao Zhang, Jishu Xian, Yongling Lu, Liang Tan, Hua Feng

**Affiliations:** 1 Department of Neurosurgery and Key Laboratory of Neurotrauma, Southwest Hospital, The Third Military Medical University (Army Military Medical University), Chongqing, China; 2 Clinical Research Center, The Third Military Medical University (Army Military Medical University), Chongqing, China

**Keywords:** artesunate, neural stem/progenitor cells, OGD, stroke, PI3K/Akt/FOXO-3a/p27 signaling pathway ^kip1^

## Abstract

Stroke is one of the leading causes of death worldwide that also result in long-term disability. Endogenous neural stem/progenitor cells (NSPCs) within subventricular (SVZ) and dentate gyrus (DG) zone, stimulated by cerebral infarction, can promote neural function recovery. However, the proliferation of eNSPCs triggered by ischemia is not enough to induce neural repair, which may contribute to the permanent disability in stroke patients. In this study, our results showed that following the treatment with artesunate (ART, 150 mg/kg), the functional recovery was significantly improved, the infarct volume was notably reduced, and the expression of Nestin, a proliferation marker of NSPCs in the infarcted cortex, was also increased. Additionally, the proliferative activity of NSPCs with or without oxygen-glucose deprivation/reperfusion was significantly promoted by ART treatment, and the therapeutic concentration was 0.8 μmol/L (without OGD/R) or 0.4 μmol/L (with OGD/R) in the *in vitro* model. Furthermore, the effects of ART can be abolished by the treatment of PI3K inhibitor wortmannin. The expression levels of related molecules in PI3K/Akt/FOXO-3a/p27^kip1^ signaling pathway (p-AKT, p-FOXO-3a, p27^kip1^) were examined using western blotting. The results suggested ART could inhibit the transcriptional function of FOXO-3a by inducing its phosphorylation, subsequently downregulating p27^kip1^ and enhancing neural stem cell proliferation in the infarcted cortex via PI3K/AKT signaling, further alleviating ischemia-reperfusion injury after ischemic stroke.

## INTRODUCTION

Cerebrovascular disease is a leading cause of disability and death worldwide, ~80% of which are ischemic stroke. Post-stroke neurological reconstruction has been widely investigated [1]. Following the occurrence of stroke, projection neurons were irreversibly injured; therefore, to replace the damaged neurons, promoting the proliferation of primary neural stem/progenitor cells (NSPCs) in a pool of stem cells in the brain using protective agents is essential [2]. The proliferation of endogenous NSPCs triggered by trauma or ischemia, is not sufficient enough to induce neural repair, which could contribute to permanent disability in stroke patients [3]. Artesunate (ART) is a water-soluble derivative of artemisinin, which has been intensively investigated due to its potential functions involved in anti-tumor [4], immune regulation [5], inhibition of inflammation [6], and treatment of type I diabetes [7]. Our previous study using an established animal model of subarachnoid hemorrhage revealed that ART could protect the blood-brain barrier by activating sphingosine 1 phosphate receptor 1/phosphatidylinositol 3 kinase (S1pR/PI3K) signaling pathway [8]. However, the effects of ART on the proliferation of NSPCs and ischemic stroke have not been elucidated.

The transcriptional factors, including FOXO, are downstream targets of PI3K/Akt signaling, which can influence the functions of FOXO by regulating its phosphorylation, consequently affecting cell growth and survival [9]. During the cell cycle, the transition from G1 to S phase needs the activation of CDK-Cyclin complexes kinase, and the activation of the Cyclin E/CDK2 complex is a key during late G1 phase [10]. The previous report has suggested that silenced FOXO3a expression in hepatic stellate cells was able to reverse arctigenin-induced upregulation of p27^kip1^, consequently promoting the activation of cyclinE-CDK2 complex, which was consistent with our findings on MCAO animals [11].

Based on these evidences, we hypothesized that ART could affect the phosphorylation of FOXO-3a, downregulate P27^kip1^ and promote the proliferation of NSPCs in the infracted cortex, subsequently alleviating ischemia-reperfusion injury after ischemic stroke through PI3K/AKT signaling.

## RESULTS

### The proliferation of NSPCs was significantly enhanced by ART

**Figure 1:**
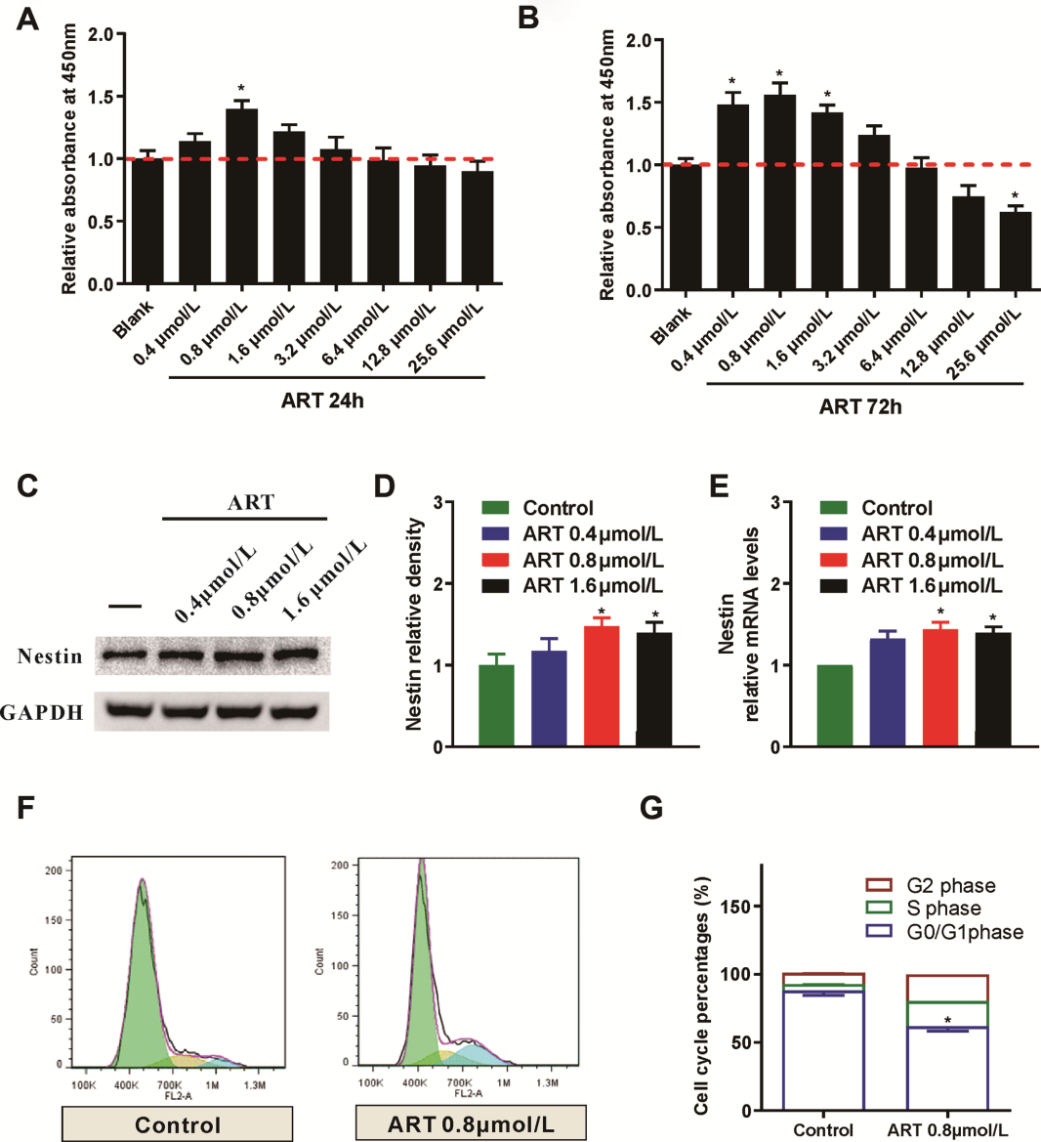
**The proliferation of NSPCs was promoted by the ART.** (**A**, **B**) CCK8 was used to examine the effects of different ART dosages (0, 0.4, 0.8, 1.6, 3.2, 6.4, 12.8 and 25.6 μmol/L) on the proliferation of neural stem cells at 24h and 72h post-treatment. (**C**, **D**) Western blotting was used to detect the expression level of Nestin in NSPCs treated with ART (0, 0.4, 0.8 and 1.6 μmol/L). (**E**) RT-qPCR was used to examine the expression of Nestin in NSPCs after the treatment. (**F**, **G**) Flow cytometry was used to evaluate the effects of 0.8 μmol/L ART on cell cycle progression of NSPCs at 72h after treatment. Data are shown as the mean ± SEM, *p< 0.05, compared to untreated group.

The effects of ART at serial concentrations on the proliferation of NSPCs were investigated. CCK8 assay was performed to screen the valid concentrations of ART at 24h (Figure 1A) and 72h (Figure 1B). The results revealed that the relative absorbance at 450nm was significantly increased in NSPCs treated with 0.4/0.8/1.6μmol/L ART compared with the control at 72h (p<0.05), and 0.8μmol/L ART exert the maximal effects, which was also observed at 24h. However, as the concentration of ART continuously increased (to 25.6μmol/L), the proliferation of NSPCs was inhibited at 72h (p<0.05).

LDH test was performed to examine the toxicity of various dosages of ART on NSPCs, and results revealed that ART concentrations showed no obvious cytotoxicity to NSCPs (p>0.05, Supplementary Figure 1). Moreover, Nestin, one type of intermediate filament protein, is a specific marker for NSPCs [12]. Our results indicated that 0.8 and 1.6 μmol/L ART significantly increased the expression of Nestin in NSPCs at 72h (p<0.05), and 0.8μmol ART exert the maximal effects (Figure 1C–1E).

**Figure 2:**
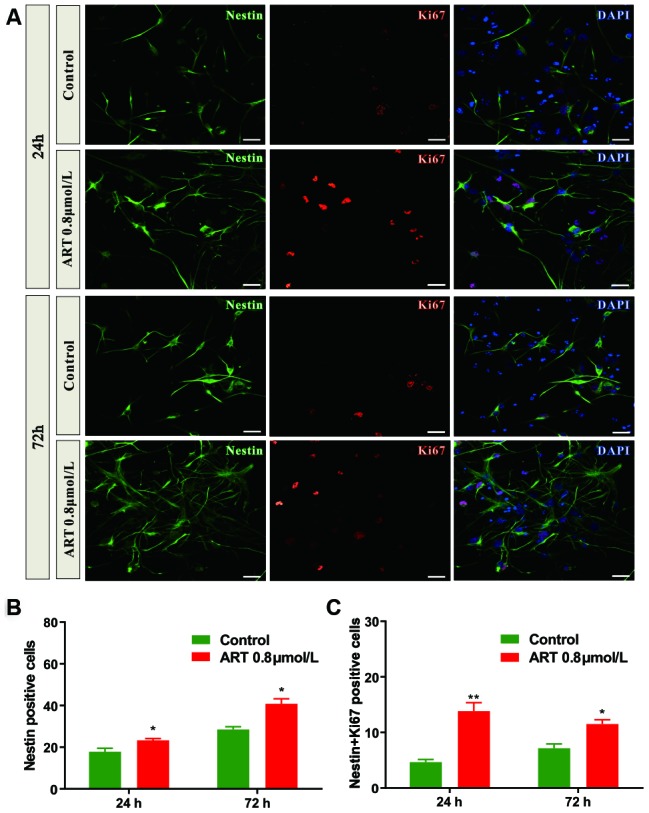
**The number of Nestin positive and/or Ki67 positive cells in NSPCs were increased by 0.8 μmol/L ART.** (**A**) Representative confocal images indicating Nestin (green) and Ki67 (red) staining in NSPCs following ART treatment (0.8μmol/L). (**B**) Quantification of the Nestin positive cells. (**C**) Quantification of the number of Nestin+Ki67 positive cells. Data are shown as the mean ± SEM. *p<0.05, **p<0.01. Scale bar = 10 μm.

Flow cytometry was performed to investigate the effects of ART on cell cycle progression. A larger proportion of NSPCs were arrested in G0/G1 phase at 72h. However, 0.8μmol/L ART-treated NSPCs exhibited a remarkably increased percentage of cells in G2/S phase compared with control (p<0.05; Figure 1F and 1G), indicating enhanced proliferative activity of NSPCs after ART treatment. As shown in immunofluorescence staining, ART treatment (0.8μmol/L) notably upregulated the number of Nestin (a neural stem cell marker) positive cells (p<0.05, Figure 2B) and the percentage of Ki67 (a proliferation marker) positive cells at 72h (p<0.05, Supplementary Figure 2). The percentage of Ki-67 positive cells among Nestin positive cells or both ki-67 and Nestin positive cells number, could clear represent the proliferation capacity of NSPCs. ART treatment also significantly increased the number of Nestin + ki67 positive cells (p<0.05, Figure 2C).

Moreover, immunofluorescence staining was also carried out to determine the effects of 0.8 μmol/L ART on the differentiation of NSPCs, especially neuronal differentiation of NSPCs. Doublecortin (DCX) is preferentially expressed in neuroblasts and as the marker of young neurons, while glial fibrillary acidic protein (GFAP) is widely used to identify astroglia in the brain. ART significantly increased the percentage of DCX+ cells (p<0.05, Supplementary Figure 3B), while reduced the percentage of GFAP+ cells (p<0.05, Supplementary Figure 3C) in in vitro. These data indicated that ART directing neuronal rather than astrocytic differentiation of NSPCs.

### The proliferation of NSPCs subjected to oxygen-glucose deprivation/reperfusion was promoted by ART

**Figure 3:**
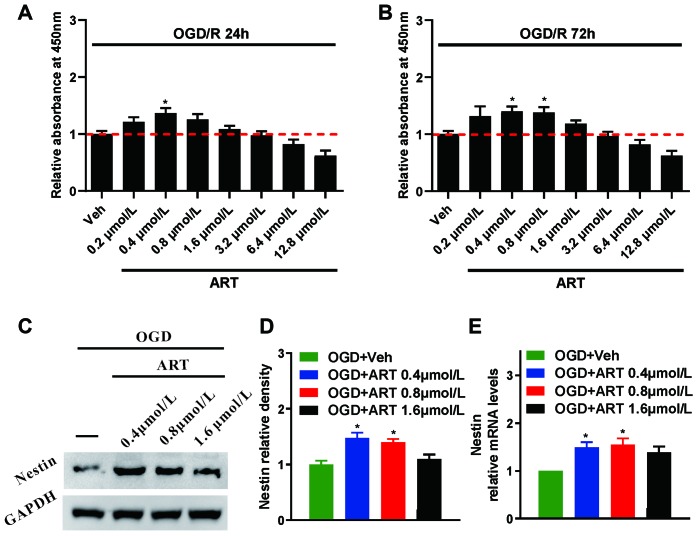
**The proliferation of NSPCs subjected to oxygen-glucose deprivation/reperfusion was improved by ART.** (**A**, **B**) After oxygen-glucose deprivation/reperfusion, CCK8 was used to examine the effects of various ART dosages (0, 0.4, 0.8, 1.6, 3.2, 6.4, 12.8 and 25.6 μmol/L) on the proliferation of NSPCs at 24h and 72h. (**C**, **D**) Western blotting was used to detect the expression of Nestin in NSPCs after oxygen-glucose deprivation/reperfusion treated with ART (0, 0.4, 0.8 and 1.6 μmol/L). (**E**) RT-qPCR was used to determine the expression level of Nestin in NSPCs treated with ART 72h after oxygen-glucose deprivation/reperfusion. Data are shown as the mean ± SEM, *p<0.05; compared to untreated group.

Oxygen-glucose deprivation/reperfusion (OGD/R) is a model used to mimic cerebral ischemia/reperfusion injury *in vitro*. After OGD/R, CCK8 was used to evaluate the effects of various ART concentrations on the proliferation of NSPCs at 24h and 72h. The results suggested that, in comparison with untreated group, the relative absorbance at 450nm was significantly increased in NSPCs treated with 0.4 μmol/L ART at 24h (p<0.05, Figure 3A), and with 0.4 μmol/L and 0.8 μmol/L ART at 72h (p<0.05, Figure 3B).

Western blot analysis (Figure 3C, 3D) and RT-qPCR (Figure 3E) were employed to determine the expression levels of Nestin in NSPCs post-OGD/R injury following the treatment with ART at various concentrations (0, 0.4, 0.8 and 1.6 μmol/L). The results indicated that 0.4 and 0.8 μmol/L ART significantly upregulated the expression of Nestin in NSPCs at 72h (p<0.05). Therefore, 0.4 μmol/L ART was used in the future experiment of the OGD/R *in vitro* model.

### PI3K/Akt/FOXO-3a/p27Kip1 signaling is involved in ART-modulated proliferation in NSPCs post-OGD/R injury

**Figure 4:**
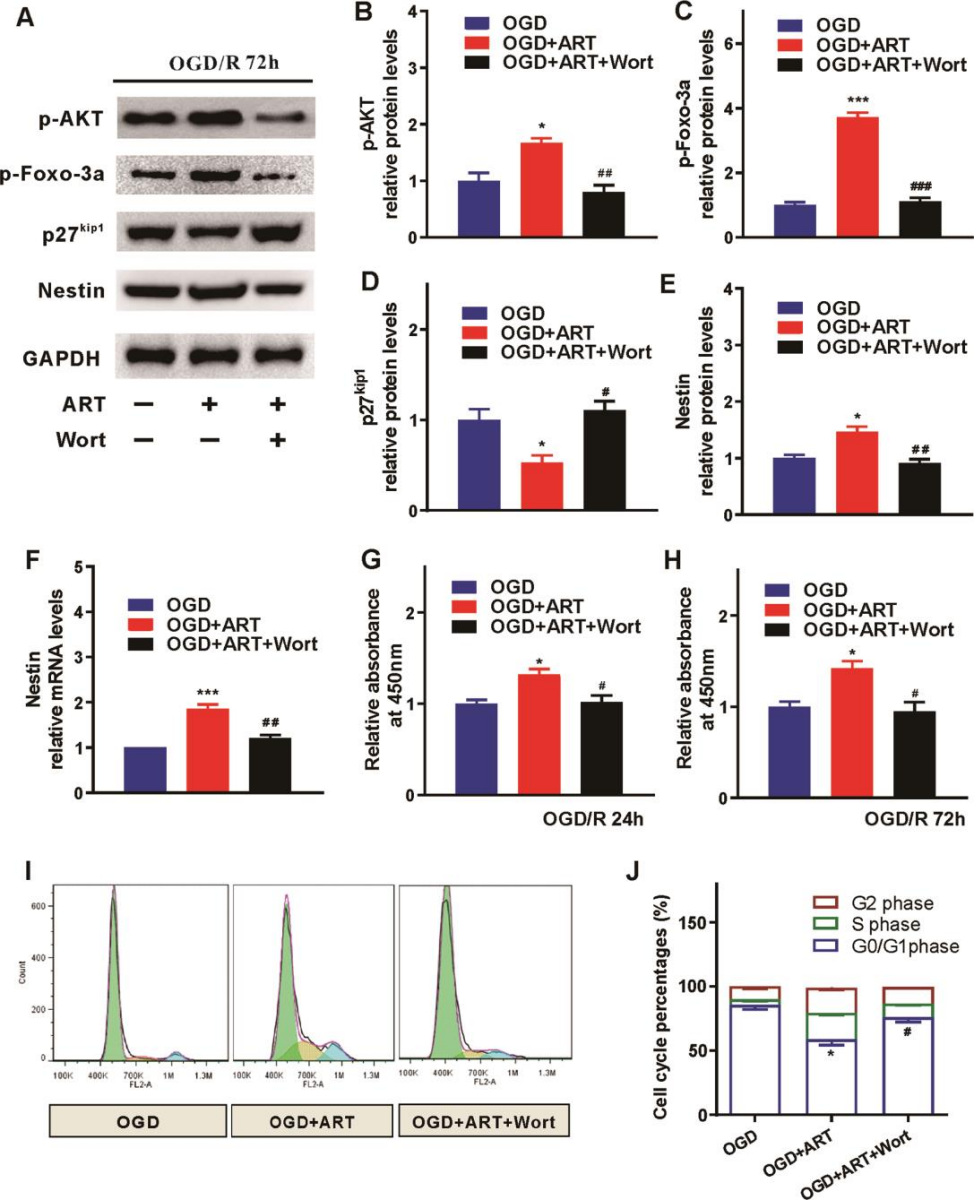
**PI3K/Akt/FOXO-3a/p27Kip1 signaling is involved in ART-induced proliferation of NSPCs after OGD/R injury.** (**A**) 72h after OGD/R, western blotting was performed in control (OGD), ART-treated (OGD+ART) and ART and wortmannin-treated (OGD+ART+Wort) group. (**B**–**E**) The expression levels of p-AKT, p-FOXO-3a, p27^kip1^, and Nestin were examined. (**F**) RT-qPCR was used to evaluate the expression of Nestin in NSPCs of different groups. (**G**, **H**) CCK8 was used to examine the proliferation of NSPCs at 24 h and 72h after OGD/R injury. (**I**, **J**) Flow cytometric analysis was carried out to determine the cell cycle progression of NSPCs 72h post-OGD/R injury. Data are shown as the mean ± SEM (*p<0.05, **p<0.01, *** p<0.001 compared to OGD group; ^#^p<0.05, ^##^p<0.01, ^###^p<0.001 compared to OGD+ART group).

To further investigate the molecular mechanisms underlying ART-induced proliferation of NSPCs after OGD/R injury *in vitro*, the effects of ART on upstream signaling cascades of FOXO-3a in the neural stem cells were studied. The FOXO transcriptional factors are downstream targets of PI3K/Akt signaling [9]. To investigate whether ART is able to inactivate FOXO-3a through PI3K/Akt pathway, 72h after OGD/R, western blotting was performed in control group (OGD), 0.4 μmol/L ART-treated group (OGD+ART), 0.4 μmol/L ART and 0.1 μmol/L PI3K inhibitor wortmannin-treated group (OGD+ART+Wort), and the expression levels of related molecules in PI3K/Akt/FOXO-3a signaling pathway (p-AKT, p-FOXO-3a), as well as Nestin, were examined (Figure 4A). ART treatment significantly promoted the phosphorylation of Akt at Ser 473 (p<0.05), whereas wortmannin notably abolished these effects (p<0.05, Figure 4B). Furthermore, ART treatment induced phosphorylation of FOXO-3a at Ser 318, and wortmannin remarkably reversed ART-mediated phosphorylation of FOXO-3a by inhibiting PI3K/Akt pathway (p<0.05, Figure 4C).

During the cell cycle, functional activation of CDK-Cyclin complexes kinase is required for the transition from G1 to S phase, which can be determined by the activation of Cyclin E/CDK2 complex during late G1 phase [10]. The inhibition of CDK2 activities may be caused by upregulated p27^Kip1^, which was modulated by transcription factor FOXO-3a. p27^Kip1^ binds with Cyclin E and reduced the amount of Cyclin E and CDK 2 in G1 phase [13]. Therefore, the expression level of these abovementioned downstream molecules (p27^Kip1^) were examined (Figure 4A). The expression of p27^Kip1^ was decreased after ART treatment, while wortmannin abrogated the effects caused by ART by inhibiting PI3K/Akt/ FOXO-3a pathway (Figure 4D).

In addition, ART-induced expression of Nestin was also reversed by wortmannin at 72h following OGD/R injury (Figure 4E, 4F). CCK8 was used to evaluate the proliferation of NSPCs at 24h and 72h after OGD/R injury. The results indicated that, in comparison with the OGD group, the relative absorbance at 450nm was significantly elevated in the OGD+ART group, but the effects of ART on NSPCs were abolished by wortmannin at both 24h and 72h after OGD/R injury (Figure 4G, 4H). Flow cytometry was used to determine the cell cycle progression of NSPCs 72h (Figure 4I) post-OGD/R injury. The enhanced proliferation of NSPCs caused by ART was significantly abrogated by wortmannin at both 24h and 72h following OGD/R injury (Figure 4J).

### Systemic administration of ART reduced motor function impairment of MCAO animals *in vivo*

The effects of various ART concentrations on motor function recovery in ischemic stroke MCAO model were investigated. There were significant decreases in survival rate and body weight compared with the sham group; however, the treatment of ART did not alleviate the mice survival rate and body weight compared with MCAO+Veh group (Supplementary Figure 4).

**Figure 5:**
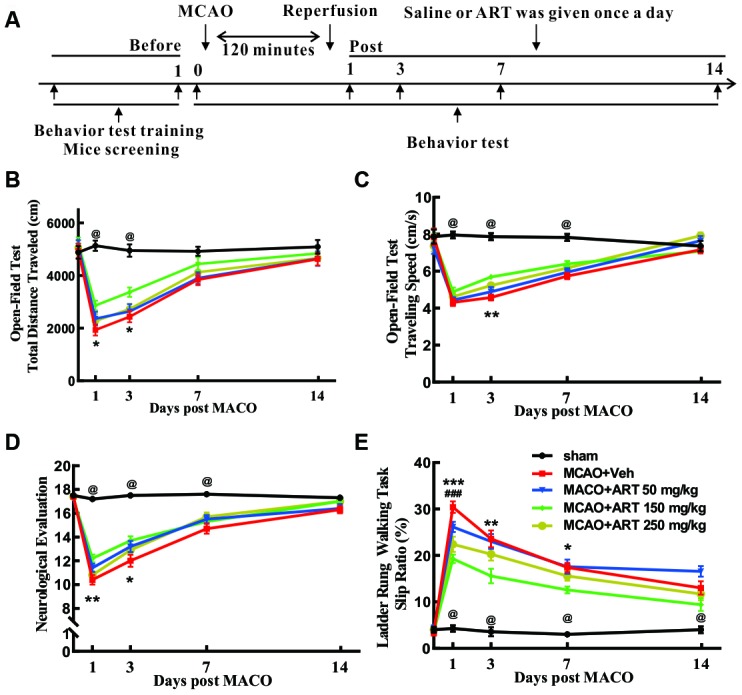
**Systemic administration of ART reduced motor function impairment of MCAO animals *in vivo*.** (**A**) The flow chart of experimental design. (**B**, **C**) The open field test was performed to measure general motor functions, and total travel distance and total travel speed were recorded. (**D**) Neurological evaluation (18-point scale assessment for the severity of impairment) was carried out to determine somatosensory movement function. (**E**) The ladder rung walking task was performed to evaluate fine motor function, and the slip ratio of the contralateral limbs was counted within 50 steps. Data are shown as the mean ± SEM, *p<0.05, **p<0.01, *** p<0.001 of MCAO+ART 150mg/kg vs. MCAO+Veh group; ^#^p <0.05, ^##^p<0.01, ^###^p<0.001 of MCAO+ART 250mg/kg vs. MCAO+Veh group; ^@^p<0.05, of MCAO+Veh group vs. sham group.

Compared with the sham group, MCAO+Veh group exhibited worse impaired performance of open-field test (total distance at 1 and 3 days after MCAO, while traveling speed at 1, 3 and 7 days after MCAO), and 150 mg/kg ART significantly improved the open-field performance (total distance traveled at 1 and 3 days after MCAO; traveling speed at 3 days after MCAO; Figure 5B, 5C). Furthermore, the MCAO+Veh group showed impaired neurological functions at 1 and 3 days after MCAO compared with the sham group, and 150 mg/kg ART notably improved the neurological evaluation at 1 and 3 days post-MCAO (Figure 5D).

The slip ratios of the ladder rung walking tasks, reflecting the fine motor function, was remarkably worse in the MCAO+Veh group from 1 to 14 days after MCAO. Surprisingly, 150 mg/kg ART rescued the slip ratios at 1, 3 and 7 days post-MCAO, and 250mg/kg ART rescued the slip ratios at day 1 following MCAO (Figure 5E).

### ART reduced the cerebral infarct volume and neurologic impairment of MCAO animals through PI3K/Akt/FOXO-3a/p27Kip1 signaling

**Figure 6:**
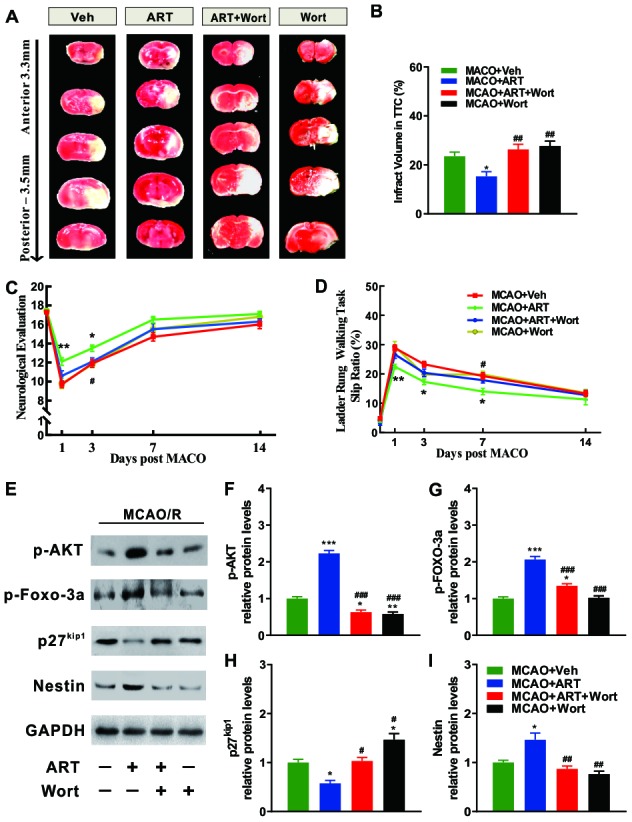
**PI3K/Akt/FOXO-3a/p27Kip1 signaling is involved in ART-mediated cerebral infarct volume and neurologic impairment of MCAO animals.** (**A**, **B**) The cerebral infarct volume was revealed by TTC staining at 72h after MCAO, and the cerebral infarct volume was quantified. (**C**) Neurological evaluation (18-point scale assessment of the impairment severity) was used to measure somatosensory movement function. (**D**) The ladder rung walking task was performed to evaluate fine motor function, and the slip ratio of contralateral limbs was counted within 50 steps. (**E–I**) Representative western blotting for PI3K/Akt/FOXO-3a signaling related molecules (p-AKT, p-FOXO-3a, p27^kip1^) and Nestin. Data are shown as the mean ± SEM. (*p<0.05, **p<0.01, *** p<0.001 vs. MCAO+Veh group; #p <0.05, ##p<0.01, ###p<0.001 vs. MCAO+ART group).

Volumes of the ischemic lesion at 72h following reperfusion demarcated from TTC staining was significantly reduced by 150 mg/kg ART (MCAO+veh vs. MCAO+ART group), whereas 1mg/kg wortmannin abolished the effects caused by ART (MCAO+ART+Wort vs. MCAO+ART group). Moreover, there was no significant difference in cerebral infarction volume between wortmannin-treated and vehicle group (MCAO+veh vs MCAO+Wort group; Figure 6A, 6B).

The performance of behavior tests was improved by ART treatment, as presented in Figure 6C, 6D, which was also abrogated by wortmannin (MCAO+ART+Wort vs. MCAO+ART group). For general somatosensory movement function, neurological evaluation revealed that the MCAO+ART group exhibited alleviated neurological impairment compared with the MCAO+Veh group at 1 and 3 days after reperfusion, and wortmannin reversed the effects caused by ART at 3 days after reperfusion (MCAO+ART+Wort vs. MCAO+ART group). Moreover, there was no significant difference in neurological evaluation between wortmannin-treated and vehicle groups (MCAO+veh vs. MCAO+Wort group, Figure 6C). For fine motor function, the ladder rung walking task suggested that the MCAO+ART group exhibited significantly reduced slip ratio of the contralateral limbs compared with the MCAO+Veh group at 1, 3 and 7 days after reperfusion, and wortmannin treatment abrogated these effects at Day 7 post-reperfusion (MCAO+ART+Wort vs. MCAO+ART group). There was no significant difference in the slip ratio between the wortmannin-treated group and vehicle group (MCAO+veh vs MCAO+Wort group, Figure 6D). These findings revealed that ART was able to improve neuro-behavioral outcomes of ischemic injury by regulating PI3K/AKT signaling pathway.

To further identify the molecular mechanisms underlying ART-modulated cerebral infarct volume and neurologic impairment in MCAO animals, the expression levels of PI3K/Akt/FOXO-3a signaling related molecules (p-AKT, p-FOXO-3a, p27^kip1^) and Nestin were examined in the infarcted cortex at 3 days post-reperfusion (Figure 6E). The phosphorylated level of Akt (p-Akt) and FOXO-3a protein (p-FOXO-3a) were elevated after the treatment with ART (150 mg/kg/day; MCAO+ART vs. MCAO+veh), and wortmannin treatment significantly abolished these effects (MCAO+ART+Wort vs. MCAO+ART group). Additionally, the expression levels of p-AKT and p-FOXO-3a in the wortmannin-treated group were reduced than the vehicle group (MCAO+vehicle vs. MCAO+Wort group, Figure 6F). The phosphorylation of FOXO-3a protein (p- FOXO-3a) was increased and the expression of P27^kip1^ was decreased after the treatment with ART (150 mg/kg/day; MCAO+ART vs. MCAO+veh), while wortmannin reversed these effects at Day 3 post-reperfusion (MCAO+ART+Wort vs. MCAO+ART group Figure 6G, 6H).

After ischemia, NSPCs become DCX+ neuroblasts before maturing into neurons and become part of functional neuronal circuits [14, 15]. Bromodeoxyuridine (BrdU) was injected intraperitoneally, to monitored the proliferation of NSPCs. The BrdU+ and DCX+ cell number was measured in ipsilateral SVZ of infarct brain. Compared to the MCAO+Veh group, the MCAO+ART group exhibited a robust increase in normalized BrdU+ and DCX+ cell number (Supplementary Figure 5A, 5B). ART (MCAO+ART vs. MCAO+veh) significantly increased the percentage of DCX+ cells, while reduced the percentage of GFAP+ cells in ipsilateral SVZ of infarct brain after MCAO (Supplementary Figure 5C, 5D). These data indicated that ART promoted post-MCAO neurogenesis and directed neuronal rather than astrocytic differentiation of NSPCs. Moreover, The expression of Nestin in the infarcted cortex was increased following the treatment with ART (MCAO+ART vs. MCAO+veh). A notable decrease was detected after wortmannin treatment (MCAO+ART+Wort vs. MCAO+ART group); however, there was no significant difference in Nestin level between MCAO+Wort and MCAO +Veh group (Figure 6I). These findings indicated that ART inhibited the transcriptional functions of FOXO-3a by promoting its phosphorylation, consequently downregulating P27kip1 and promoting neurogenesis in the infarcted cortex through PI3K/AKT signaling pathway.

## DISCUSSION

ART is a water-soluble derivative of artemisinin, which penetrates the blood-brain barrier easily. It exhibits anti-malarial functions with high-efficiency and low-toxicity. ART is widely used in clinical practice and is recommended by the World Health Organization for the treatment of malaria, especially for cerebral and falciparum malaria [16]. ART has been intensively investigated due to its potential functions involved in anti-tumor [4], immune regulation [5], inhibition of inflammation [6], and treatment of type I diabetes [7]. An animal model of subarachnoid hemorrhage has been established in our previous study, and the data revealed that ART could protect the blood-brain barrier by activating sphingosine 1 phosphate receptor 1/phosphatidylinositol 3 kinase (S1pR/PI3K) signaling pathway [8].

Acute ischemic stroke is characterized by severe physical, cognitive and psychiatric impairments, which can result in disability and mortality. Intriguing, a slow but consistent functional recovery from stroke-induced neuro-dysfunction was observed in clinical practices and animal models over weeks and months [2, 17]. The neurogenesis initiated by neural endogenous stem cells serves essential roles in this process. Under normal physiological conditions, NSPCs present in the SVZ area and migrate to the olfactory bulb, where they become granule and periglomerular neurons, while the newly formed neuroblasts in the SGZ migrate and become dentate granule cells in dentate gyrus, which maintain and reorganize the existing circuitry, consequently affecting memory and behavior [1, 18]. During ischemia, NSPCs accelerate its proliferation and differentiation and then migrate to the surrounding infarction, where they differentiate into mature neurons or glial cells, further becoming a part of functional neuronal circuits [14]. The proliferation of endogenous neural stem cells, triggered by trauma or ischemia, is not sufficient enough to induce neural repair, which could contribute to the permanent disability of stroke patients [3]. Due to impaired blood-brain barrier, excitotoxicity and neuroinflammation, the damaged brain exhibits notably disrupted cellular microenvironment that is hostile to the survival of NSPCs [19, 20].

To improve ischemic stroke-induced neuro-recovery, enhanced proliferation and differentiation of NSPCs could be a novel therapeutic approach. In the present study, the proliferation of NSPCs was remarkably promoted by ART treatment at relatively low concentrations. These findings indicated that the cell cycle arrest of NSPCs at the G0/G1 phase could be disturbed by ART (Figures 1, 2). Moreover, ART promoted the proliferation of NSPCs subjected to OGD/R, suggesting that ART may protect the NSPCs from OGD/R-induced injury (Figure 3). The expression level of Nestin was increased following the treatment with ART, suggesting the proliferative activity of NSPCs in the infarcted cortex was enhanced (Figure 6). The abovementioned results indicate that ART can promote the proliferation of NSPCs under both physiological and ischemic conditions. In vitro, ART significantly increased the percentage of DCX+ cells, while reduced the percentage of GFAP+ cells in vitro (Supplementary Figure 3), indicating that ART directing neuronal rather than astrocytic differentiation of NSPCs. LDH test was also performed to examine the toxicity of ART on NSPCs, and results indicated that the concentrations used in this study exhibited no obvious cytotoxicity on NSCPs; however, as the concentration increased, 25.6μmol/L ART inhibited the proliferation of NSPCs, suggesting the toxicity of high dosage of ART and further experiments are required.

The proliferation of NSCPs and subsequent neurogenesis are involved in injury alleviation and functional recovery following various brain injuries [1, 20]. To evaluate the effects of ART on alleviating injury and promoting functional recovery of ischemic stroke, the MCAO mice model was established. The results revealed that 150 mg/kg ART significantly reduced the infracted brain volume (Figure 6), neurogenesis (Supplementary Figure 5, Figure 6I) and improved motor function impairment (Figure 5).

Furthermore, the mechanism of ART-induced proliferation of NSPCs and neuroprotective effects after OGD/R and MCAO injury had not been completely elucidated. Several genetic studies in mammalian revealed that FOXO transcription factors (FOXO 1, 3 and 4) act as critical downstream regulators of PI3K/Akt pathway and regulate numerous genes involved in various cellular processes such as proliferation, survival, metabolism, differentiation and cell cycle [21]. As revealed by the study on immortal Hydra, upregulation of FOXO in stem cell lineages is crucial for the continuous self-renewal, and silenced FOXO in epithelial cells promotes its terminal differentiation into foot cells [22]. Paik JH reported that mammalian FOXOs enhance the stability of long-lived cells such as thymocytes, endothelial cells and hematopoietic stem cells (HSCs) [23]. FOXO-3a is a key factor in cell resistance to external stimuli by binding to DNA and exerting its nuclear transcriptional roles, further participating in various biological functions [24]. FOXO-3a can be phosphorylated by a variety of upstream molecules, such as Akt. When NSPCs are stimulated, FOXO3a is dephosphorylated and transferred to the nucleus to induce multiple gene transcriptional regulation, such as p27^Kip1^ [11]. CDK and cyclin are the key molecules involved in DNA synthesis, chromosome separation and cell division [25]. The kinase activities of CDK-Cyclin complexes, that regulate the process of cell cycle, could be inhibited by cyclin-dependent kinase inhibitors (CKIs), including the INK4 protein family and kinase inhibiting protein (KIP) family [26]. The activity of protein kinase complex CyclinE-CDK2 could be downregulated by p27^Kip1^, which suppresses cell cycle progression from G0/G1 to S phase [27]. The previous report has revealed that silenced FOXO3a expression in hepatic stellate cells reversed the upregulation of p27^Kip1^ caused by arctigenin, subsequently promoting the formation of CyclinE-CDK2 complexes and triggering G0/G1 phase cell cycle arrest, which was consistent with our findings using MCAO animals [28]. Phosphorylated Akt was considered as an essential transcription factor of the FOXO family such as FOXO1/FKHR, FOXO3a/FKHRL1, and FOXO4/AFX; PI3K and its downstream molecule Akt can regulate the entry of cell cycle by inactivating FOXO, which stimulates the expression of quiescence genes including p27^Kip1^ [29]. And our data revealed that ART inhibited the transcriptional function of FOXO-3a by promoting its phosphorylation, downregulated P27^kip1^ and promoted neural stem cell proliferation in the infarcted cortex through PI3K/Akt signaling pathway, subsequently alleviating injury and improving functional recovery of ischemic stroke (Figures 4, 6).

There are several limitations in this study. Use of a transgenic labeling approach would be better to support the conclusion of this study. There could be numerous variables, especially side effects in other organs and systems, which should be investigated in the future prior to clinical trials. To motor function outcome, the MCAO rodents got significant improvement even one day after ART treatment, which could be related to other potential functions of ART. According to our previous study, ART could protect the blood-brain barrier by activating sphingosine 1 phosphate receptor 1/phosphatidylinositol 3 kinase (S1pR/PI3K) signaling pathway, and artesunate alleviated neurologic impairment and brain edema 24h after an established animal model of subarachnoid hemorrhage [8]. For blood-brain barrier preservation plays an important role in attenuating injury after MCAO, we speculate that the ART could protect the blood-brain barrier after MCAO. On the other hand, ART can attenuate inflammatory responses. ART activates Nrf2 pathway-driven anti-inflammatory potential and attenuates LPS-induced inflammatory responses in microglial BV2 cells [30]. Also, Artesunate attenuates lipopolysaccharide-stimulated proinflammatory responses by suppressing TLR4, MyD88 expression, and NF-kappa B activation in microglial cells [31]. For attenuating acute inflammatory responses plays an important role in alleviating injury after MCAO, we speculate that the ART attenuates acute inflammatory responses to improve motor function outcome even 1 day after ART treatment. However, further experiments are needed.

**Figure 7:**
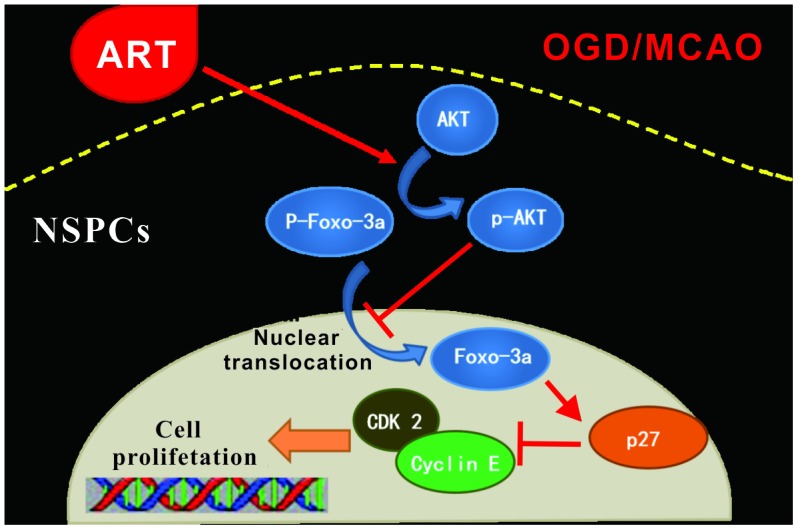
**Schematic summary.** ART is able to promote the proliferation of NSPCs after ischemic injury both *in vivo* and *in vitro*, through the AKT/FOXO-3a/p27Kip1 pathway, thus improve the ischemic penumbra and reduce infarct volume, further promoting functional reconstruction.

In conclusion, cellular and animal models were established to demonstrate that ART inhibited cell cycle arrest of stem cells in a dose-dependent manner and promoted the proliferation of NSPCs by inducing the phosphorylation of FOXO-3a via PI3K/Akt pathway, subsequently reducing the infracted brain volume and alleviating motor function impairment caused by MCAO (Figure 7). These findings improved our understanding of the molecular mechanisms underlying NSPCs proliferation in post-stroke spontaneous recovery, suggesting that ART could be a potential therapeutic agent for the treatment of ischemic stroke.

## MATERIALS AND METHODS

### Establishment of mouse middle cerebral artery occlusion/reperfusion model

The animal study was approved by the Animal Care Committee of the Third Military Medical University (Army Medical University; no. SYXK 2012-0002). A total of 21 newborn and 120 adult male C57BL/6 mice were purchased from the Animal Center of the Third Military Medical University. This study was performed in accordance with the China animal welfare legislation for scientific purposes.

The establishment of the model of middle cerebral artery occlusion (MCAO) was carried out as previously described [32]. Briefly, C57BL/6 mice were anesthetized using 2% isoflurane/air mixture (1-2 L/min). A 2.0-cm silicone-coated 8-0 nylon suture (Sunbio Biotech Co., Ltd., Beijing, China) was gently inserted from the external carotid artery stump to the internal carotid artery, which terminated at the opening of middle cerebral artery. Therefore, focal cerebral ischemia was induced by transient monofilament occlusion of the right middle cerebral artery occlusion for 90 minutes before the cerebral blood flow was restored, and reperfusion was performed by withdrawal of the inserted filament. Sham-operated mice received the same surgical procedures without occlusion. All surgical operations were performed in a sterilized environment with maintained temperature at 37±0.5°C, and the mice accessed to food and water freely post-surgery. After the mice were recovered using a 4-point neurological deficit severity scale [32], neurological deficits were graded, and MCAO mice scored 2-3 were randomly grouped.

### Cells culture and establishment of OGD/R model

Primary neural stem/progenitor cells (NSPCs) were cultured as previously described [33, 34]. Briefly, cells were cultured using Dulbecco's Modified Eagle’s Medium (DMEM) containing 10% fetal bovine serum (FBS, vol/vol, Hyclone, Logan, Utah). The cortex was washed twice, and then treated using 0.25% trypsin-EDTA (Hyclone, Logan, Utah) for at 37°C 30 minutes. The tissue samples were rinsed twice using DMEM and then triturated using a fire-polished Pasteur pipette and passed through a 100-μm Nylon cell strainer (BD Falcon, San Jose, CA). The cells were maintained in DMEM/Nutrient Mixture F-12 (DMEM/F12) medium supplemented with B27 (GIBCO, Grand Island, NY), 20 ng/ml EGF (Peprotech, Rocky Hill, NJ) and 20 ng/ml FGF-2 (Peprotech, Rocky Hill, NJ) in a humidified atmosphere with 5% CO_2_ at 37°C. For the passaged cells, the neurospheres were harvested by centrifugation (300 rpm), dissociated using StemPro Accutase cell dissociation reagent (GIBCO, Grand Island, NY), and cultured in the abovementioned medium. The NSPCs used in this study were passages 3-5.

To induce ischemic insult *in vitro*, the OGD/R model was established as previously described [12]. Briefly, cells were incubated with glucose-free and FBS-free Earle's BSS buffer (Thermo Forma, Thermo Fisher Scientific, Boston, MA, USA). The cells were then immediately transferred into a humidified anaerobic chamber supplemented with 94% N_2_, 5% CO_2_ and 1% O_2_ at 37°C for 4 h. During the OGD, intervention drugs were added into the medium accordingly. To terminate cell OGD and perfusion, the medium was replenished by DMEM/F12 supplemented with B27 (GIBCO, Grand Island, NY), 20 ng/ml EGF (Peprotech, Rocky Hill, NJ) and 20 ng/ml FGF-2 (Peprotech, Rocky Hill, NJ), under the recommended conditions.

### Experimental design and drug treatment

### Experiment 1

To evaluate the effects of ART (Holley-Wulingshan pharmaceuticals Corp. Ltd., Chongqing, China) at serial concentrations on the proliferation of NSPCs, cells were seeded onto 96-well plates at a density of 5x10^4^/well and incubated with ART at the concentrations of 0, 0.4, 0.8, 1.6, 3.2, 6.4, 12.8 and 25.6 μmol/L for 24 h and 72 hours, and the cell culture medium added with ART was changed once a day. Then, the CCK8 assay was performed. To further determine the effects of ART, western blotting and RT-qPCR were also performed to examine the expression levels of Nestin in NSPCs treated with ART (0, 0.4, 0.8 and 1.6 μmol/L) for 72 hours. In addition, flow cytometry by PI stained cells was used to evaluate the effects of 0.8 μmol/L ART on cell cycle progression of NSPCs for 72 hours. Immunofluorescence staining was carried out to determine the expression of Nestin and Ki67 in NSPCs treated with 0.8 μmol/L ART. Furthermore, the LDH test was performed to examine the toxicity of this dosage of ART on NSPCs. Immunofluorescence staining was also carried out to determine the effects of 0.8 μmol/L ART on the differentiation of NSPCs.

### Experiment 2

To investigate the effects of various concentrations of ART on the proliferation of NSPCs following oxygen-glucose deprivation/reperfusion. NSPCs were seeded onto 96-well plates and subjected to oxygen-glucose deprivation/reperfusion at a density of 5x10^4^/well, then incubated with ART at serial concentrations of 0, 0.2, 0.4, 0.8, 1.6, 3.2, 6.4 and 12.8 μmol/L for 24h and 72h, and the cell culture medium added with ART was changed once a day. Afterward, the CCK8 assay was performed. To further evaluate the effects of different concentrations of ART on the proliferation of NSPCs subjected to oxygen-glucose deprivation/reperfusion, western blot analysis and RT-qPCR were performed to determine the expression levels of Nestin in NSPCs treated with ART at various doses (0, 0.4, 0.8 and 1.6 μmol/L) for 72 hours.

### Experiment 3

To further study the effects of ART on the proliferation of NSPCs and the underlying mechanisms after OGD/R injury *in vitro*, NSPCs were randomly assigned into three groups: control group (OGD), 0.4 μmol/L ART-treated group (OGD+ART), 0.4 μmol/L ART- and 0.1 μmol/L PI3K inhibitor wortmannin-treated group (OGD+ART+Wort). The cell culture medium added with ART was changed once a day. CCK8 assay was used to examine the proliferation of NSPCs at 24h (I) and 72h (J) post-OGD/R injury. Additionally, western blotting was performed to detect PI3K/Akt/FOXO-3a signaling related molecules and Nestin in NSPCs at 72h after OGD/R injury. RT-qPCR was carried out to determine the expression levels of Nestin in NSPCs 72h post-OGD/R injury. Furthermore, flow cytometric analysis was used to identify the roles of PI3K/Akt/FOXO-3a signaling on ART-modulated cell cycle progression of NSPCs for 72h.

### Experiment 4

To evaluate the effects of different concentrations of ART on motor function recovery in ischemic stroke MCAO model, MCAO mice were randomly assigned into four groups: MACO+Veh group (equal dose of saline); MACO+50 mg/kg ART group; MACO+150 mg/kg ART group; MACO+250 mg/kg ART group. Together with the sham group, there were five groups in total. The drug was administered intraperitoneally on a daily basis, and according to our previous findings, the first dose was given immediately following reperfusion. The functional behavioral test was performed, including the open field Test, neurological evaluation, and the ladder rung walking task.

### Experiment 5

In order to further study the effects of ART on ischemic injury *in vivo* and investigate the novel neuroprotective roles of ART, MCAO mice were randomly divided into four groups: MACO+Veh group; MACO+150mg/kg ART group (MCAO+ART group); MACO+150mg/kg ART + 1 mg/kg wortmannin group (MCAO+ART+Wort group) and MACO+1 mg/kg wortmannin group (MCAO+Wort group). The drug was administered intraperitoneally once per day and the first dose was given immediately after reperfusion. 2, 3, 5-triphenyltetrazolium Hydrochloride (TTC) Staining was carried out to examine the cerebral infarcted volume at 72h post-surgery. Neurological evaluation and the ladder rung walking task were used to evaluate behavior performance. Immunofluorescence staining was used to detect the neurogenesis of NSPCs in SVZ following MCAO. Western blotting was performed to determine the expression levels of PI3K/Akt/FOXO-3a/p27^kip1^ signaling-related molecules and neural stem cell marker in the infarcted cortex at 72h after MCAO.

### CCK-8 assay

The NSPCs were seeded onto 96-well plates at a density of 5×10^3^/well, and received different intervention for 24h and 72h. Then, 10 μl of CCK8 reagent (Solarbio Science and Technology Co., Ltd., Beijing, China) was added to each well and incubated at 37°C for 2h. The absorbance was detected at 450 nm using a microplate reader (Thermo Scientific, Finland). Relative absorbance was calculated as: OD450 of treated well/OD450 of blank sample.

### LDH test

Lactate dehydrogenase (LDH) releasing test was performed to examine the toxicity of various dosages of ART on NSPCs. 100 μl of the NSPCs single cell suspension (~10,000 cells) was seeded onto a 96-well plate and cultured for three days, and then LDH cytotoxicity test kit (Beijing Solarbio Science and Technology Co., Ltd., China) was used according to the manufacturer’s protocols. LDH releasing reagent provided within the kit was added to the positive control group (10% of the volume of the original culture medium). After the addition of the LDH releasing reagent, the mixture was repeated pipetting several times to mix, followed by incubation for 1 hour. After that, the plate was centrifuged at 400 g for 5 mins. Then, 120 μl of the supernatant was taken from each well and added onto a fresh 96-well plate, and 60 μl of LDH detection working solution was added to each well. The plate was mixed and incubated at room temperature for 30 mins in the dark. The absorbance was then measured at a wavelength of 490nm. The absorbance at 600 nm was used as a reference.

### Western blotting

Isolated NSPCs or brain tissues were homogenized, and then total proteins were extracted using a protein extraction kit (Beyotime Biotechnology, Co. Ltd., China). Equal amount of extracted proteins was loaded onto 10% SDS-polyacrylamide gels. The proteins were separated and then transferred onto PVDF membranes (Millipore Inc., Billerica, MA, US). The membranes were blocked with 3% bovine serum album (BSA) in TBST at room temperature for 2 hours, and then incubated using rabbit polyclonal antibody against Nestin (Abcam, 1:2000), rabbit polyclonal antibody against FOXO-3a (R&D, 1:1000), rabbit polyclonal antibody against phosphorylated FOXO 3a (Ser 318, R&D, 1:1000), mouse monoclonal antibody against p27^kip1^ (CST, 1:2000), rabbit polyclonal antibody against Cyclin E (CST, 1:1000), rabbit polyclonal antibody against CDK2 (CST, 1:1000), rabbit polyclonal antibody against AKT (Abcam, 1:2000), rabbit polyclonal antibody against phosphorylated Akt (Ser 473, Abcam, 1:2000), or mouse monoclonal antibody against GAPDH (Zsgb-bio, 1:1000) at 4°C overnight. After being incubated with corresponding peroxidase-conjugated secondary antibodies (Zsgb-bio, 1:5000) for at room temperature 2h, the signals were detected using ChemiDoc™ XRS + imaging system by Pierce fast western blot kit (Thermo Scientific, US). GAPDH, an internal control, was used to normalize the expression level of each protein. Protein bands were quantified using densitometry with Quantity One software (Bio-Rad Laboratories, Inc., Hercules, CA, US) [35].

### RNA extraction and RT-qPCR

Total RNA of hippocampi and cortices (n=24) were extracted using Trizol RNA extraction kit (Takara), and the concentrations of extracted RNA samples were determined (Thermo Hybaid, Germa). Reverse transcription was performed using PrimeScript™ RT reagent Kit with gDNA Eraser according to the manufacturer’s protocols (Takara). Specific primers of Nestin and GAPDH were designed according to the RNA sequences obtained from Gene Bank. Forward and reverse PCR primer sequences were as followed: Nestin, 5’-AGGAGAAGCAGGGTCTACAGAG-3’ and 5’-AGTTCTCAGCCTCCAGCAGAGT-3’; and GAPDH, 5’-CATCACTGCCACCCAGAAGACTG-3’ and 5’-ATGCCAGTGAGCTTCCCGTTCAG-3’.

### Flow cytometry

To examine cell cycle distribution, the NSPCs were seeded onto culture dishes, then incubated for 24h to allow exponential growth and treated with ART at indicated concentrations for 72h. Following the addition of 10ml of 70% ethanol, the collected cells were stored at -20°C for 24h. After three washes using cold phosphate buffered saline (PBS), the samples were resuspended in 500μl propidium iodide (PI)/Triton X-100 staining solution and incubated at 20°C for 30 min. The fluorescence was measured using FACS Caliber flow cytometer (BD, San Jose, CA, USA). Flow cytometric data were analyzed by FlowJo software (version 10, FlowJo, LLC, Ashland, Oregon, USA). Forward scatter (FSC) versus side scatter (SSC) plots were gated in order to exclude cellular debris. And RCS (or RMSD) less than 0.8 or greater than 5.0 are considered unacceptable.

### Bromodeoxyuridine (BrdU) injection and incorporation assay

The BrdU incorporation assay was performed as previously described [36]. To analyze the proliferation of neuroblasts, 100mg/kg of BrdU was administrated intraperitoneally every 24h, and the first dose was given immediately after surgery. Mice were sacrificed at 3 days post-MCAO, and coronal sections (25μm in thickness) were stained for BrdU-incorporated DCX+ cells. The co-staining of BrdU+ and DCX+ cells was observed on confocal images. Cell counting was performed on four slices per brain to calculate the relative percentage of cells present in two areas.

### Immunofluorescence staining

Following the treatment, NSPCs or brain tissues were fixed using 4% paraformaldehyde at room temperature for 1h. After being blocked in 1% BSA, 5% goat serum and 0.2% Triton X-100 in PBS (PH 7.4) at 4°C for 2h, the specimens were incubated with with corresponding antibodies against against Ki67(1:1000, Abcam, UK), DCX (1:500, Abcam, UK), BrdU (1:500, Millipore, Germany), GFAP(1:500, Abcam, UK), Nestin (1:1000, Proteintech Group, Inc, US) at 4°C overnight. Then, cells were incubated with corresponding secondary antibody, goat anti-rabbit Alexa Fluor 488, goat anti-rabbit Alexa Fluor 555, goat anti-rabbit Alexa Fluor 647, Goat Anti-Rat Cy3 or anti-mouse Alexa Fluor 488 (1:200, Beyotime, China) at room temperature for 2h, followed by counterstaining with 4,6-diamidino-2-phenylindole dihydrochloride (DAPI, 2 mg/ml in PBS) for 10 min. Finally, the images were captured using a Zeiss UV 780 Meta confocal microscope (Carl Zeiss) and examined by Adobe Photoshop CS6 software.

For BrdU immunostaining, brain sections were incubated in 2N HCl at 37°C for 30min. Stained sections were rinsed using 0.1M borate solution (pH=8.5) twice for 10min, incubated in 3% H_2_O_2_ for 30 min and blocked with 5% normal goat serum at room temperature for 1h.

### Functional behavioral test

Neurological evaluation was performed to examine the somatosensory movement function as previously described. Briefly, the evaluation was perform in a double-blind manner, and using an 18-point scoring method. Open-field test was also performed, as previously reported, by using the open-field activity system and software (Noldus Information Technology Co, Ltd), [37–39].

The ladder-rung walking task, which reveals the subtle loss of movement capacity, was also conducted as previously described [40]. All animals were trained to walk on the ladder rungs 1 day before MCAO. Mice with <50 steps after MCAO were excluded. The number of correct placement of contralateral forelimb and hind-limb within 50 steps were counted [38]. All experiments and analyses were performed by individuals blinded to the treatment groups.

### 2, 3, 5-triphenyltetrazolium hydrochloride (TTC) staining and measurement of infarct volume

At 24h and 72h post-reperfusion, animals were sacrificed by common carotid perfusion fixation with cold Tris-buffered saline under isoflurane anesthesia. The brain was immediately removed and sectioned into five coronal slices (2mm in thickness) using a brain-cutting matrix. The slices were then stained with 1% 2, 3, 5-triphenyltetrazolium chloride (TTC; Sigma-Aldrich Pty Ltd, Australia) at 37°C in the dark for 30 min. Non-infarcted tissues were stained (red) and infarct tissues were not stained (white). The percentage of infarct volume was analysed using Image J software 1.50i by calculating the infarct volume ratio. Briefly, the infarct volume was calculated as a percentage of the entire brain adjusted for edema using modified Swanson calculation [41].

### Statistical analysis

All data were presented as mean ± standard error of the mean (SEM). Student’s t test or one-way ANOVA with post-hoc LSD test was used for comparisons, as appropriate. Behavioral data collected at corresponding time points were analyzed using two-way repeated-measures ANOVA, followed by the Bonferroni post-hoc test. Statistical significance was two-tailed, and *p*<0.05 indicated statistically significant difference. The results were shown as the mean values of at least three independent experiments.

## Supplementary Material

Supplementary Figures
