# Supplementary material for: Artesunate promotes the proliferation of neural stem/progenitor cells and alleviates Ischemia-reperfusion Injury through PI3K/Akt/FOXO-3a/p27kip1 signaling pathway
Source: Aging (Albany NY). 2020 May 7;12(9):8029–48. doi: 10.18632/aging.103121 (PMC7244066; doi:10.18632/aging.103121)
Supplement: Supplementary Figures [file aging-12-103121-s001..pdf]

SUPPLEMENTARY FIGURES

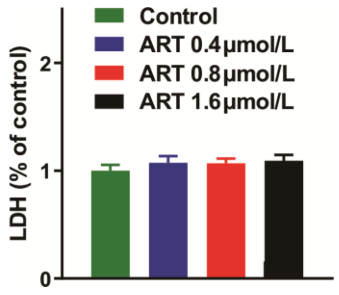

Supplementary Figure 1. LDH assay was employed to detect cytotoxicity of ART on NSPCs. Data are presented as the mean ± SEM (*p<0.05). ART: artesunate.

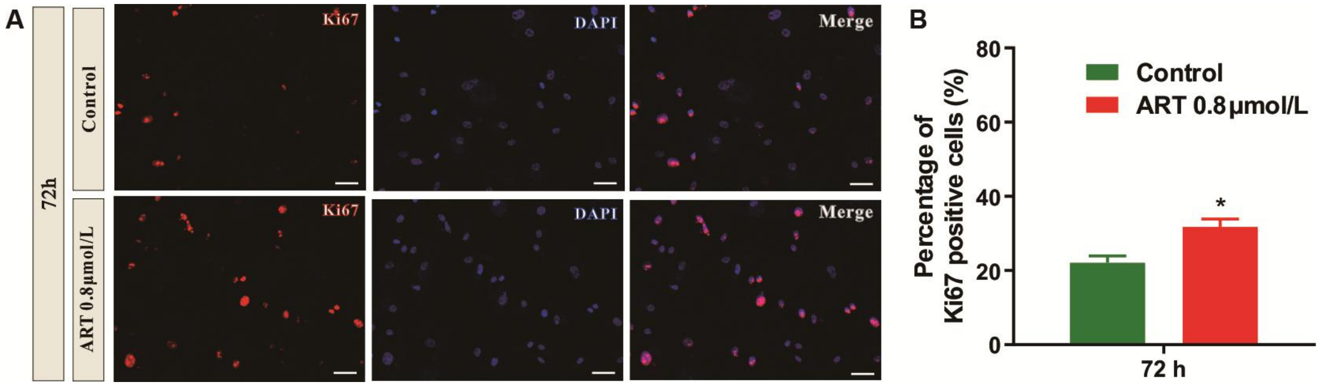

Supplementary Figure 2. The percentage of ki67 positive cells was increased by 0.8 μmol/L ART. (**A**) Representative confocal images indicating DAPI (blue) and Ki67 (red) staining in NSPCs following ART treatment (0.8μmol/L). (**B**) Quantification of the ratio of ki67 positive cells after 72h. Data are shown as the mean ± SEM. *p<0.05. Scale bar = 15 μm. ART: artesunate.

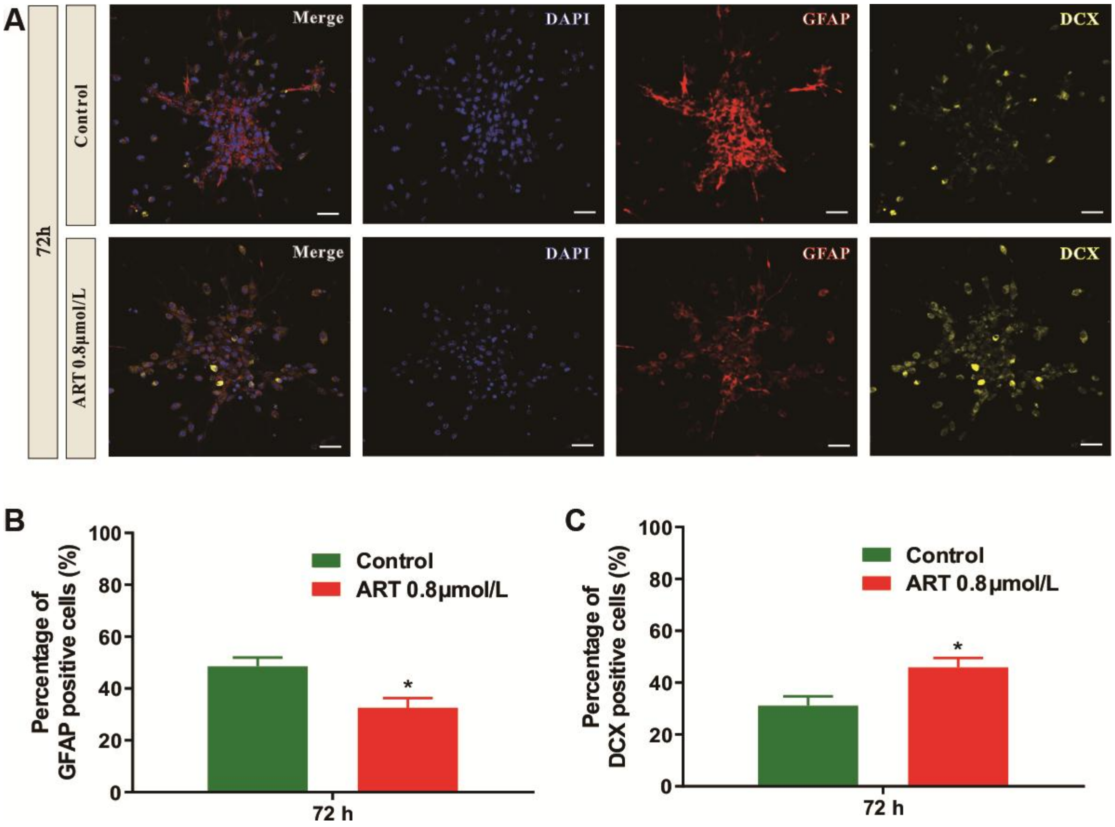

Supplementary Figure 3. ART induced preferred neuronal differentiation of NSPCs in vitro. (**A**) Representative immunostaining of DAPI+, DCX+ and GFAP+ (Scale bar=15 μm). (**B**) Quantitative analyses of the Percentage of GFAP+ cells. (**C**) Quantitative analyses of the percentage of DCX+ cells. *p<0.05. ART: artesunate.

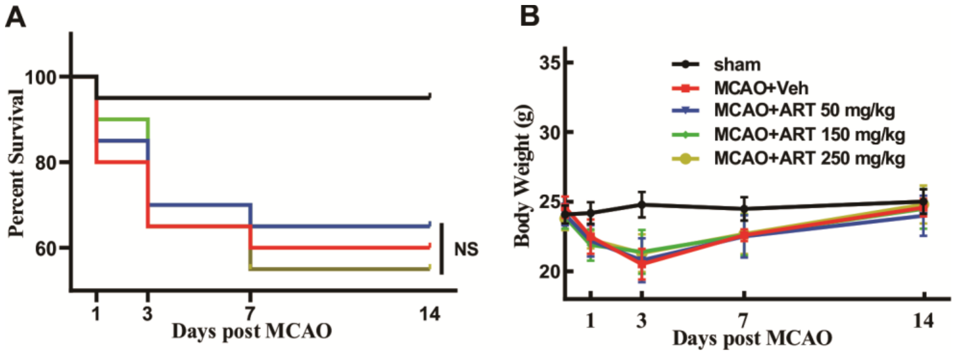

Supplementary Figure 4. Systemic administration of ART exhibits no effects on mice survival rate and body weight. (**A**) Survival rate at 0, 1, 3, 7, and 14 days post-MCAO. (**B**) Bodyweight changes measured at 0, 1, 3, 7, and 14 days following MCAO. Data are shown as the mean ± SEM,*p<0.05. ART: artesunate.

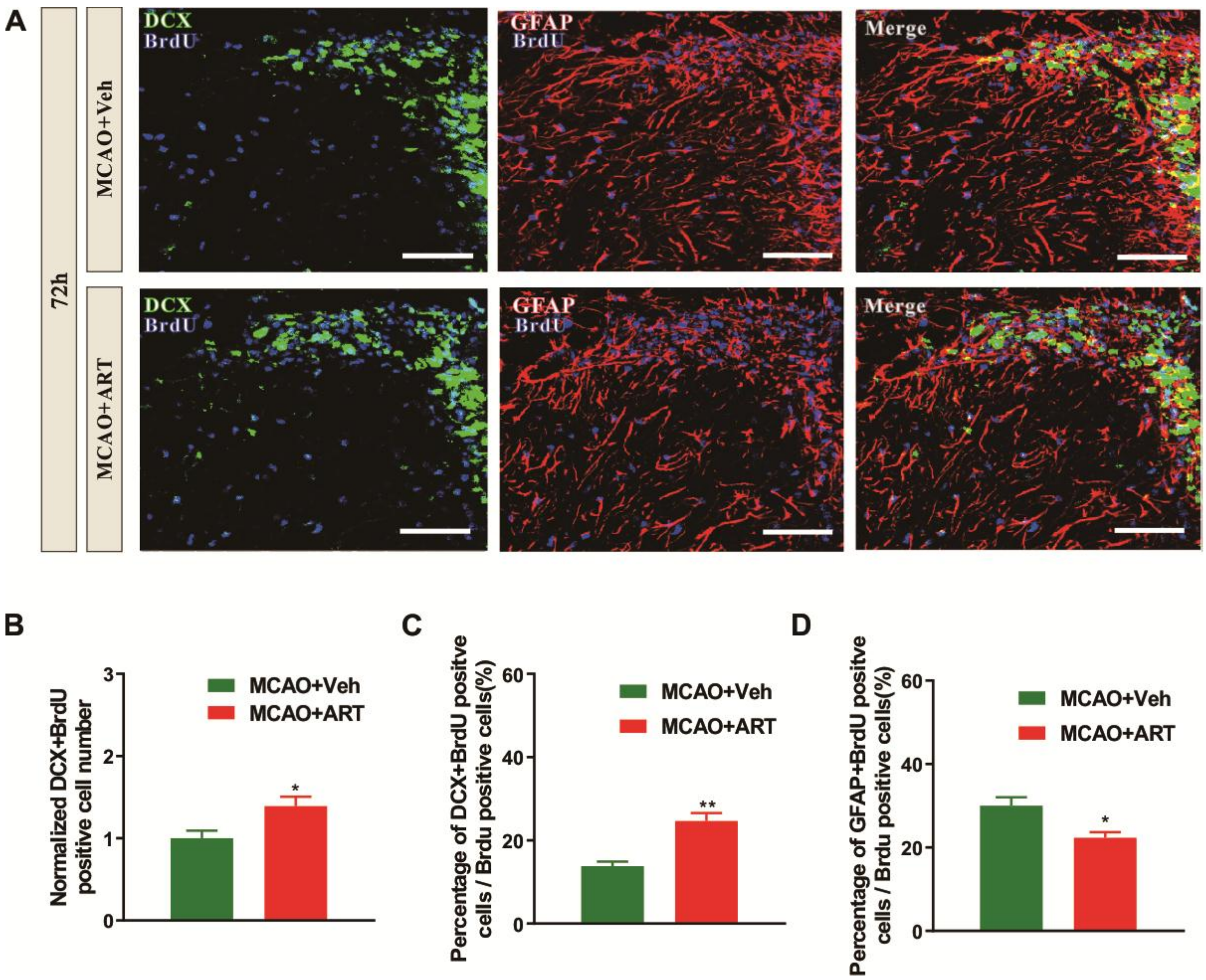

Supplementary Figure 5. ART promoted neurogenesis after MCAO. (**A**) Representative immunostaining of BrdU+, DCX+ and or GFAP+ in SVZ 3 days after MCAO (Scale bar=100 μm). (**B**) Quantitative analyses of the normalized numbers of DCX+ and BrdU+ cells. (**C**) Quantitative analyses of Percentage of DCX+BrdU positve cells/BrdU positive cells. (**D**) Quantitative analyses of Percentage of GFAP+BrdU positve cells/BrdU positive cells. *p<0.05, **p<0.01 vs. MCAO+Veh group. ART: artesunate.
